# Role of polarized cell divisions in zebrafish neural tube formation

**DOI:** 10.1016/j.conb.2009.04.010

**Published:** 2009-04

**Authors:** Jon Clarke

**Affiliations:** MRC Centre for Developmental Neurobiology, King's College London, New Hunt's House, 4th Floor, Guy's Hospital Campus, SE1 1UL London, United Kingdom

## Abstract

Development of epithelial cell polarity and morphogenesis of a central lumen are essential prerequisites for the formation of the vertebrate neural tube. In teleost fish embryos this first involves the formation of a solid neural rod structure that then undergoes a process of cavitation to form a lumen. This process is initiated from a neural plate that has a distinct organization compared to other vertebrates, and involves complex cell intercalations and rearrangements. A key element is a mode of polarized cell division that generates daughters with mirror-image apico-basal polarity. These mirror-symmetric divisions have powerful morphogenetic influence because when they occur in ectopic locations they orchestrate the development of ectopic apical and basal specializations and the development of ectopic neural tubes.

At early stages in development the vertebrate neural tube is a fairly simple pseudostratified epithelium that surrounds a central lumen. The apical or lumenal side of the early epithelium is specialized for proliferation and the basal or pial side is specialized for differentiation of the post-mitotic neurons and elaboration of their dendritic and axonal processes. A single continuous central lumen contains the choroid plexus that secretes the cerebrospinal fluid that circulates to bathe both the internal and external surfaces of the neural tube. Generation of an organized epithelial structure and a coherent central lumen are thus major steps in the morphogenesis of the vertebrate central nervous system. This process is probably best understood in birds and mammals and since excellent, recent and comprehensive reviews of neural tube formation in these animals are available elsewhere (see, for example, references [1–3]) this process will not be dealt with in detail here. The key points relevant to the following discussion are that the initial structure of the amniote neural plate is already organized as a single cell layered columnar epithelium with clear apical (superficial) and basal (deep) polarity. Apico-basal polarity of the amniote neural plate is not generated at the neural plate stage, but rather is inherited from the already polarized structure of the epiblast. Neural induction triggers a thickening of the epiblast in the region of the neural plate as the epithelial cells become taller, but the basic epithelial polarity was already in place. This relatively simple epithelium is then transformed into a neural tube by longitudinal folding mechanisms that essentially roll up the lateral edges of the flat neural plate until they meet and fuse together to form a tube with a single continuous central lumen. This process is called primary neurulation.

In contrast to amniotes, another major vertebrate group – the teleosts – generate their neural tubes by a rather different mechanism, one that forms a central lumen not by folding an epithelial sheet but by first building a solid neural rod primordium that then undergoes a rearrangement of its cells to ‘cavitate’ and form a hollow tube [4,5]. Until recently the cellular and molecular details of teleost neurulation have however been missing, but this is now changing with the emergence of the zebrafish embryo as arguably the premier vertebrate model system for studying the regulation of morphogenesis. The following discussion and opinion will centre on new work that has addressed neural tube morphogenesis in the zebrafish embryo. It will pay particular attention to the role of cell divisions in this process as recent results suggest the novel possibility that divisions orchestrate both the polarization of cells and the cell rearrangements required to generate a central lumen.

## Cell organization in zebrafish neural plate

The structure of the zebrafish neural plate is rather poorly understood with several recent publications presenting conflicting views of its organization. It has variously been described as a single cell-layered columnar epithelium [6,7,8^•^,9^••^], a bilayered structure with similarities to the organization of the Xenopus neural plate [10] and a multilayered structure composed of multipolar, largely spheroidal cells [11^••^]. A comprehensive analysis of neural plate organization at different antero-posterior levels and at different developmental stages is required to resolve these differences, but perhaps the most likely explanation of these varying views is that the organization is very dynamic and differs along the antero-posterior axis. Our own [11^••^] work suggests that in the anterior regions of the neural plate (prospective forebrain, midbrain and hindbrain) the neural plate is a multilayered structure three to six cells deep, which in the anterior spinal cord region gradually thins down to become a single cell thickness in the more posterior regions. In the prospective hindbrain region very few, if any, plate cells have the typical elongated spindle morphology characteristic of more mature neuroepithelial cells although some scattered cells are somewhat elongated across the superficial deep axis. Thus, at this stage the hindbrain neural plate is a stratified structure, but unlike a mature neuroepithelium not yet pseudostratified. Also although several cell layers deep, distinct layers are not obvious. It could be argued that many anterior neural plate cells may, in fact, have very thin processes that are difficult to resolve with light microscopic techniques but do connect them to either the deep or the superficial surface of the plate. If this were the case then their organization may not be so dissimilar to the amniote neural plate, but this issue awaits more detailed analysis. The analysis of polarity proteins however also suggests organization the fish neural plate is quite distinct from that of amniotes.

## The zebrafish neural plate is not a conventional epithelium

Analyses with markers of apico-basal polarity demonstrate that the fish neural plate is not a conventional epithelium [8^•^,10]. Although the deep surface of this tissue sits on a basal lamina, conventionally apical proteins such as aPKC and ZO-1 are not restricted to the superficial surface but are either unpolarized or randomly scattered close to plasma membranes throughout the depth of the plate.

For most vertebrate embryos, the organization of the neural plate not only provides the basic structure of the neuroepithelium but also forms part of the continuous protective epithelial ectoderm layer that surrounds the whole embryo. In the zebrafish this protective role for the neural plate is not necessary as this role is taken by the enveloping layer (EVL, [12]). The EVL is a monolayer of simple squamous epithelial cells that completely envelops the embryo from the end of gastrulation onwards. The EVL lies immediately above and very close to the superficial surface of the neural plate but remains essentially motionless throughout the neural plate's movements of convergent extension, invagination and cavitation to form the neural keel, rod and then tube. In this way the zebrafish neural plate can be considered free to adopt the non-epithelial organization that allows the more complex cell rearrangements and polarizations that transform plate through keel, rod and then neural tube.

**Figure 1:**
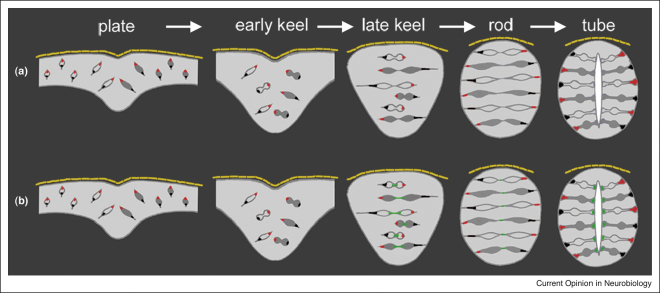
**(a)** Diagrammatic transverse sections to illustrate cell organization and behaviour that transform the neural plate through the stages of neural keel, rod and then neural tube. At all stages the neural primordium is covered by the overlying simple squamous epithelium of the enveloping layer (EVL, shown in yellow). Cells derived from the left hand side of the neural plate are shown in outline and cells derived from the right hand side are shown filled. The superficial pole of cells in the plate is marked with a red dot and the deep pole of these cells is marked with a black dot. Following the red dots through the process of the midline crossing division reveals that the superficial pole of plate cells is destined to become the basal extremity of the daughter that crosses the midline, and the black dots reveal that the deep pole of plate cells will become the basal extremity of the ipsilateral daughter. **(b)** The distribution of the apical protein Pard3-GFP (shown in green) has been added to illustrate the development of mirror-image apical polarity. In the late keel and rod stages Pard3-GFP is found accumulating at the cleavage furrow and close to the midbody of cells undergoing the C-division. Pard3-GFP remains around this location as the cells complete cytokinesis and is thus mirror-symmetrically inherited into the prospective apical ends of the daughter cells that go on to form the endfeet that line the lumenal surface of the neural tube.

The lack of obvious apical polarity in the neural plate may be best understood in relation to the behaviour and fate of these cells during the transition from neural plate to neural tube. This transition is characterized by a highly stereotyped cell division that almost all neural plate cells seem to undergo. Neural plate cells converge to the dorsal midline where they complete a mediolaterally oriented cell division that deposits one daughter cell on the left hand side of the neural keel or rod and the other daughter on the right hand side [7,8^•^,13^•^]. Because one daughter crosses the midline we have called this division the C-division (for crossing-division, [11^••^]). The daughter that stays on its side of origin is always the one closest to its ipsilateral basal surface (and may be connected to this surface by a thin cellular process throughout the C-division, [9^••^]) and the crossing daughter is always the one closest to the midline. The side of this crossing daughter that lies closest to the midline will then stretch across the contralateral side of the neural rod to contact the opposite basal surface. Topologically this prospective basal process originated from the superficial side of the neural plate (Figure 1). Thus, although the deep surface of the neural plate is the prospective basal surface of the ipsilateral neural tube, the superficial surface of the plate does not equate to the prospective apical surface. In fact because of the midline crossing division the side of a cell that is closest to the superficial surface of the zebrafish neural plate will initially be relocated to the centre of the neural keel but (following the C-division) will then intercalate through the contralateral side of the neural rod to establish contact with the contralateral basal surface. Thus, the superficial side of cells in the neural plate is in fact prospective basal for the contralateral side of the neural tube. It seems unlikely that this superficial surface will be molecularly specified as basal, but that is its eventual fate.

Although the scattered distribution of apical proteins such as aPKC and ZO-1 in the neural plate [8^•^] confirm that an apical plane is not established at these stages, the dynamic behaviour of these cells suggest they are almost certainly polarized across the superficial to deep axis of the plate in some way. For example, as cells converge to the midline they elongate across the superficial to deep axis of the neural plate [10], and in the hindbrain region of the plate when these cells divide their daughters also separate across the superficial to deep axis [11^••^]. These behaviours suggest cells are able to detect the superficial to deep axis of the plate, and a potential source of molecular cues to this axis could be the mesoderm that underlies the neural plate or a basal lamina that most probably lies between neural plate and mesoderm.

## Cell division directs cell polarization in the neural keel and rod

During late neural keel and neural rod stages, cells are predominantly oriented horizontally across the mediolateral axis and many cell processes and cell bodies lie across the midline [9^••^,10,11^••^]. In order to transform this midline into a lumen, cells must re-arrange so as not to lie across the midline and they must polarize to generate apical or lumenal membrane along this midline plane. This rearrangement and polarization appears to be orchestrated by the C-division itself. The C-division is a mirror-symmetrically polarized division because it generates daughters with mirror-image apico-basal polarity and morphology. Interestingly the generation of mirror-image apical polarity seems to be established before the completion of cytokinesis as a GFP tagged version of the apical protein Pard3 localizes to the cleavage furrow or either side of the abscission plane of the C-division and each daughter then inherits and maintains Pard3-GFP at this medial location [11^••^].

The power of the mirror-symmetric C-division to organize the cell rearrangements and polarizations required for lumen formation is illustrated in mutants with ectopic C-divisions. In embryos with defective Wnt/Planar Cell Polarity (PCP) signalling mirror-symmetric C-divisions occur ectopically on either side of the midline instead of at the midline itself. These ectopic C-divisions divide along the superficial deep axis of the neural plate and generate mirror-symmetric pairs of daughters that lie along this axis too. Apical protein is deposited along the plane of cleavage of these ectopic divisions, and this generates an ectopic apical plane and subsequently ectopic lumens. Mirror-symmetric neuroepithelial organization forms on either side of these ectopic lumens [11^••^]. Pharmacological block of these ectopic divisions eliminates the generation of these ectopic lumens thus demonstrating they are generated by the divisions rather than some other defect of planar cell polarity.

The mirror-image polarity of the zebrafish C-division may be a very specialized case of a general property of cell divisions. For example, when *Dictyostelium* cells divide they mirror-symmetrically localize the phosphoinositide signalling regulators PI3K and PTEN. PI3 kinase is localized to the distal poles of dividing cells and its antagonistic tumour suppressor PTEN is localized to the cleavage furrow [14]. This may be particularly relevant to the zebrafish C-division as PTEN has also been implicated in the organization of lumenal membrane in MDCK cysts in culture [15], and PTEN associates with Bazooka (the Drosophila homologue of Pard3) at the apical membrane of fly epithelia and neuroblasts [16]. Dividing 3T3 cells have also been described as generating daughters with mirror-symmetric morphology, cytoskeletal patterns and even migratory behaviour [17], and most recently Caco-2 cells in a 3-dimensional culture assay have been shown to localize the apical proteins aPKC and ZO-1 to the cleavage furrow or midbody of dividing cells during cyst formation [18]. The mechanisms that tie apical polarization and lumen formation to cytokinesis have yet to be worked out, but one common theme to each of these three processes in other systems is membrane traffic. Apical polarization (reviewed in [19]), lumen formation (reviewed in [20]) and cytokinesis (reviewed in [21]) all depend on specific membrane trafficking events, so perhaps this common subcellular process is harnessed by the C-division to efficiently generate and localize apical specializations.

## Radial and contralateral cell intercalation

The transformation of the anterior multilayered neural plate to the pseudostratified epithelium of the neural tube requires cell interdigitation and intercalation across the layers. This process occurs progressively as the neural plate undergoes convergence movements to the midline and invagination at the midline to form the neural keel [10]. Cells gradually elongate and interdigitate across the superficial deep axis of the neural plate as it converges to the midline and then the superficial to deep axis and with it the axis of interdigitation rotates through 90° during invagination. Thus, in the keel intercalation occurs across the mediolateral axis. The cell adhesion molecule N-Cadherin may play a key role in interdigitation and intercalation as N-Cadherin mutants generate a complex neuroepithelial organization that time-lapse analysis suggests could result from defective interdigitation of superficial and deep layers [10]. However, since zebrafish N-Cadherin is also required to complete convergence of the lateral neural plate [10,22] another interpretation of the N-Cadherin mutant data is that the ectopic tissue planes that accumulate apical junctional proteins such as aPKC and ZO-1 (see Figure 9 in [10]) result from ectopic mirror-symmetric C-divisions in the lateral domains of the neural plate. Ectopic C-divisions might be expected there because the convergence defect is similar to (but more severe than) that in Wnt/PCP defective embryos that have been shown to generate ectopic C-divisions [11^••^]. This would explain why the junctional proteins are located in a plane sandwiched between the superficial and deep surfaces of the non-converged lateral neural plate rather than at the superficial surface, and would imply this plane is a nascent apical and lumenal surface. However, the organization of the ectopic junctional plane in N-Cadherin mutants is much less ordered than in embryos with convergence defects due to either defective planar cell polarity signalling or surgical intervention [11^••^] thus suggesting N-Cadherin function is more complex than just a requirement for convergence of the lateral neural plate. Apart from convergence this additional function could be in the establishment and maintenance of an organized apical surface [22–25] or as suggested by Hong and Brewster [10] in regulating cell intercalation between superficial and deep cells.

## Contralateral intercalation

It could be suggested that the behaviour of the daughter cell that crosses the midline after the C-division and integrates into the contralateral neural rod is a special case of radial intercalation and thus might be regulated by specific mechanisms. One prerequisite for integration into the contralateral side is that progenitors from the two sides must adhere and maintain contact. A recent study shows that the receptor tyrosine kinase EphA4 and its ligand EfnB2a independently mediate this cell affinity in alternating segments of the hindbrain [26]. Furthermore, the alternating segmental expression of these membrane proteins appears to ensure that intercalation across the midline only occurs between cells of the same segmental identity, thus, maintaining an accurate alignment of segments across the midline [26].

An earlier study has also implicated the Wnt/PCP pathway in the intercalation of daughter cells into the contralateral side of the neural keel and rod. A central core of ectopic cells is found in the neural tube of embryos with severe disruption of the Wnt/PCP pathway suggesting contralateral integration is compromised [9^••^]. While some of the data presented in this study (see, for example, Figures 2f and g, and 3g in [9^••^]) are reminiscent of the ectopic mirror-image neuroepithelia described by Tawk *et al*. [11^••^] other images do suggest that a more disorganized central core of ectopic cells can be generated. Ciruna *et al*. [9^••^] make the interesting proposal that in the absence of PCP signalling these ectopic cells have lost the polarization signals required to intercalate into the contralateral side of the neural rod. Furthermore, by analysing the distribution of a Prickle-GFP fusion protein they suggest the crucial cues lost by these cells may be cues of antero-posterior polarity rather than mediolateral polarity as might be expected. Understanding the differences between some of the phenotypes seen in the Ciruna *et al*. [9^••^] and Tawk *et al*. [11^••^] papers will take further investigation, but could be due to different mechanisms having varying levels of importance at different levels of the antero-posterior axis (spinal cord versus hindbrain), or due to differing severity of the genetic defects. Both studies, however, reveal key roles for both the C-division and Wnt/PCP signalling in morphogenesis of the zebrafish neural tube. The Tawk *et al* [11^••^] study suggests that, in line with its role in mice and Xenopus (reviewed in [2,3,27]), the principal role of PCP signalling is to regulate the efficiency of convergence of the neural plate to the midline. This is slower in PCP defective embryos and this results in ectopic C-divisions that then drive ectopic lumen formation. The Ciruna *et al*. [9^••^] study postulates that in addition to regulating convergence, PCP signalling is also required for the cell polarization needed for intercalation of the daughter cells of the C-division into the contralateral side of the neural rod.

## Conclusions

The mirror-symmetric C-division is a very stereotyped cell behaviour—it occurs within a small window of time with a strict mediolateral orientation and in a very specific location close to the midline of the neural keel and rod. Since ectopic C-divisions can have severe consequences on neural tube morphogenesis its occurrence must be tightly regulated in the embryo. Future experiments should address the mechanisms that control this division—is it regulated by local environmental cues from within the neural primordium or from neighbouring tissues, or could it be controlled by an intrinsic timing programme? Mechanisms controlling cell polarization, cell intercalation and cell sorting are also likely to be crucial regulators of this morphogenesis and are likely to have general relevance to other examples of lumen formation during organogenesis, including perhaps the process of secondary neurulation that also depends on generating a lumen from a solid neural primordium and makes the most caudal segments of spinal cord in most vertebrates including man.

## References and recommended reading

Papers of particular interest, published within the period of review, have been highlighted as:

• of special interest
•• of outstanding interest

## References

[1] Colas J.F., Schoenwolf G.C. (2001). Towards a cellular and molecular understanding of neurulation. Dev Dyn.

[2] Copp A.J., Greene N.D., Murdoch J.N. (2003). The genetic basis of mammalian neurulation. Nat Rev Genet.

[3] Ueno N., Greene N.D. (2003). Planar cell polarity genes and neural tube closure. Birth Defects Res C Embryo Today.

[4] Kunz Y.W. (2004). Developmental Biology of Teleost Fishes.

[5] Lowery L.A., Sive H. (2004). Strategies of vertebrate neurulation and a re-evaluation of teleost neural tube formation. Mech Dev.

[6] Schmitz B., Papan C., Campos-Ortega J.A. (1993). Neurulation in the anterior trunk region of the zebrafish *Bracydanio rerio*. Roux's Arch Dev Biol.

[7] Papan C., Campos-Ortega J.A. (1994). On the formation of the neural keel and neural tube in the zebrafish Danio (Brachydanio) rerio. Roux's Arch Dev Biol.

[8•] Geldmacher-Voss B., Reugels A.M., Pauls S., Campos-Ortega J.A. (2003). A 90-degree rotation of the mitotic spindle changes the orientation of mitoses of zebrafish neuroepithelial cells. Development.

[9••] Ciruna B., Jenny A., Lee D., Mlodzik M., Schier A.F. (2006). Planar cell polarity signalling couples cell division and morphogenesis during neurulation. Nature.

[10] Hong E., Brewster R. (2006). N-cadherin is required for the polarized cell behaviors that drive neurulation in the zebrafish. Development.

[11••] Tawk M., Araya C., Lyons D.A., Reugels A.M., Girdler G.C., Bayley P.R., Hyde D.R., Tada M., Clarke J.D. (2007). A mirror-symmetric cell division that orchestrates neuroepithelial morphogenesis. Nature.

[12] Sagerström C.G., Gammill L.S., Veale R., Sive H. (2005). Specification of the enveloping layer and lack of autoneuralization in zebrafish embryonic explants. Dev Dyn.

[13•] Kimmel C.B., Warga R.M., Kane D.A. (1994). Cell cycles and clonal strings during formation of the zebrafish central nervous system. Development.

[14] Janetopoulos C., Borleis J., Vazquez F., Iijima M., Devreotes P. (2005). Temporal and spatial regulation of phosphoinositide signaling mediates cytokinesis. Dev Cell.

[15] Martin-Belmonte F., Gassama A., Datta A., Yu W., Rescher U., Gerke V., Mostov K. (2007). PTEN-mediated apical segregation of phosphoinositides controls epithelial morphogenesis through Cdc42. Cell.

[16] von Stein W., Ramrath A., Grimm A., Müller-Borg M., Wodarz A. (2005). Direct association of Bazooka/PAR-3 with the lipid phosphatase PTEN reveals a link between the PAR/aPKC complex and phosphoinositide signaling. Development.

[17] Albrecht-Buehler G. (1977). Daughter 3T3 cells. Are they mirror images of each other?. J Cell Biol.

[18] Jaffe A.B., Kaji N., Durgan J., Hall A (2008). Cdc42 controls spindle orientation to position the apical surface during epithelial morphogenesis. J Cell Biol.

[19] Mostov K., Su T., ter Beest M. (2003). Polarized epithelial membrane traffic conservation and plasticity. Nat Cell Biol.

[20] Lubarsky B., Krasnow M.A. (2003). Tube morphogenesis, making and shaping biological tubes. Cell.

[21] Montagnac G., Echard A., Chavrier P. (2008). Endocytic traffic in animal cell cytokinesis. Curr Opin Cell Biol.

[22] Lele Z., Folchert A., Concha M., Rauch G.J., Geisler R., Rosa F., Wilson S.W., Hammerschmidt M., Bally-Cuif L. (2002). parachute/n-cadherin is required for morphogenesis and maintained integrity of the zebrafish neural tube. Development.

[23] Bronner-Fraser M., Wolf J.J., Murray B.A. (1992). Effects of antibodies against N-cadherin and N-CAM on the cranial neural crest and neural tube. Dev Biol.

[24] Radice G.L., Rayburn H., Matsunami H., Knudsen K.A., Takeichi M., Hynes R.O. (1997). Developmental defects in mouse embryos lacking N-cadherin. Dev Biol.

[25] Ganzler-Odenthal S.I., Redies C. (1998). Blocking N-cadherin function disrupts the epithelial structure of differentiating neural tissue in the embryonic chicken brain. J Neurosci.

[26] Kemp H.A., Cooke J.E., Moens C.B. (2008). EphA4 and EfnB2a maintain rhombomere coherence by independently regulating intercalation of progenitor cells in the zebrafish neural keel. Dev Biol.

[27] Doudney K., Stanier P. (2005). Epithelial cell polarity genes are required for neural tube closure. Am J Med Genet C Semin Med Genet.

